# Biosynthesized Silver Nanoparticles by Aqueous Stem Extract of *Entada spiralis* and Screening of Their Biomedical Activity

**DOI:** 10.3389/fchem.2020.00620

**Published:** 2020-08-19

**Authors:** Wan Khaima Azira Wan Mat Khalir, Kamyar Shameli, Seyed Davoud Jazayeri, Nor Azizi Othman, Nurfatehah Wahyuny Che Jusoh, Norazian Mohd Hassan

**Affiliations:** ^1^Department of Chemical and Environmental Engineering, Malaysia-Japan International Institute of Technology, Universiti Teknologi Malaysia, Kuala Lumpur, Malaysia; ^2^Centre for Virus and Vaccine Research, School of Science and Technology, Sunway University, Subang Jaya, Malaysia; ^3^Kulliyyah of Pharmacy, International Islamic University Malaysia, Kuantan, Malaysia

**Keywords:** silver nanoparticles, biosynthesis, *Entada spiralis*, antibacterial assay, physicochemical parameters

## Abstract

Silver nanoparticles (Ag-NPs) have been established as antibacterial nanoparticles and have been innovatively developed to overcome the occurrence of antibiotic resistance in the environment. In this study, an environmentally friendly and easy method of the biosynthesis of Ag-NPs plants, mediated by aqueous extract stem extract of *Entada spiralis* (*E. spiralis*), was successfully developed. The *E. spiralis*/Ag-NPs samples were characterized using spectroscopy and the microscopic technique of UV-visible (UV-vis), X-ray Diffraction (XRD), Field Emission Transmission Electron Microscope (FETEM), zeta potential, and Fourier Transform Infrared (FTIR) analyses. Surface Plasmon Resonance (SPR) absorption at 400–450 nm in the UV-vis spectra established the formation of *E. spiralis*/Ag-NPs. The crystalline structure of *E. spiralis*/Ag-NPs was displayed in the XRD analysis. The small size, around 18.49 ± 4.23 nm, and spherical shape of Ag-NPs with good distribution was observed in the FETEM image. The best physicochemical parameters on Ag-NPs biosynthesis using *E. spiralis* extract occurred at a moderate temperature (~52.0°C), 0.100 M of silver nitrate, 2.50 g of *E. spiralis* dosage and 600 min of stirring reaction time. The antibacterial activity was tested against *Staphylococcus aureus, Enterococcus faecalis, Escherichia coli*, and *Proteus vulgaris* using an antibacterial disk diffusion assay. Based on the results, it is evident that *E. spiralis*/Ag-NPs are susceptible to all the bacteria and has promising potential to be applied in both the industry and medical fields.

## Introduction

The related applications based on nanotechnology, are in great demand nowadays due to the unique biological, electrical, and optical biological properties of metal nanoparticles. Properties such as antibacterial, antifungal, anticancer, antioxidant, wound healing ability, coloration, conductivity, UV blocking, photocatalytic, and self-cleaning activity provide the materials with different functions (Wang et al., 2017; Keshvadi et al., 2019). These nanoparticle properties are widely used in biomedical areas, health care, drug-gene delivery, wound healing, cosmetics, textiles, environmental pollutants, electrical appliances, non-linear optical devices, and photo-electro chemicals. The nanotechnology design process involves controlling, measuring, and producing materials on a nano scale of ~1–100 nm. The type of nanoparticles currently used for any application by industries are gold (Au), magnetite (Fe_3_O_4_), Titanium oxide (TnO_2_), Zinc oxide (ZnO), and Copper oxide (CuO) (Nava et al., 2016; Reddy, 2017). Among these metal nanoparticles produced today, silver (Ag) is one of the most popular and valued metals—especially in consumer products and in the medical field (Shameli et al., 2012; Khatoon et al., 2017).

Ag-NPs have antibacterial properties and are well-known to be an effective disinfectant against a wide range of microorganisms and can also treat bacterial infections through a longer time of exposure due to its good stability (Li et al., 2016). Almost 650 microorganisms including Gram-positive bacteria and Gram-negative bacteria, fungi, and viruses are shown to have antimicrobial activity against Ag-NPs (Ahmed et al., 2016). This positive result makes Ag-NPs a potential material to be applied in many applications such as in the medical field, packaging material, environmental pollution, textile industries, fabric, coating of biomaterials, tissue engineering, cancer diagnosis and treatments where antibacterial agents are crucially needed (Nassar and Youssef, 2012; Chouhan et al., 2017; Narasaiah et al., 2018). The exact mechanisms by which Ag-NPs kill bacteria remain a challenge for most researchers. However, predictions by some researchers on possible mechanisms, could be due to the contact action, the release of Ag^+^ ions and generation of reactive oxygen species (Wu et al., 2018). Most of the listed applications of Ag-NPs deal with human contact. Therefore, it is compulsory to develop an environmentally friendly synthesis method which eliminates or minimizes the use of toxic chemicals that can affect human health and the environment. The bottom-up methods that use biosynthesis mediated from plant extract are considered to be an environmentally friendly method. This method has caught the attention of researchers due to its ease, biodegradability, biocompatibility, natural abundance, good nanoparticle distribution, small sized nanoparticles, stability in colloidal forms, its low cost, mild reaction, and also, its minimal use of hazardous materials during the synthesis process (Mittal et al., 2013).

The morphology, stability, particle size distribution, and surface charge of metal nanoparticles play a very significant role in the controlled synthesis of Ag-NPs using plant extract as a reductant and stabilizing agent (Rajakumar et al., 2017). These can be controlled by varying the physicochemical parameters such as the initial concentration of silver nitrate, stirring time, and plant dosage (Dwivedi and Gopal, 2010; Polte, 2015; Wu et al., 2018). Besides that, the antibacterial activity performance also depends on the size of Ag-NPs and the gram character of the bacteria. Nowadays, the susceptibility of Ag-NPs toward Gram-positive and Gram-negative bacteria continues to be debated in this research area. A study by Ravichandran et al. (2016) reported that the average size of Ag-NPs obtained is 38 nm using an extract concentration of 1.5 mL of 10% *Artocarpus altilis* leaf extract, 1.0 mM of silver nitrate, reaction time of 60 min, pH 7, and a temperature of 70°C. The prepared Ag-NPs showed that *Escherichia coli* and *Pseudomonas aeruginosa* are more susceptible to Ag-NPs than *Staphylococcus aureus*. In another study, Yan-yu et al. (2016) conducted the biosynthesis of Ag-NPs using *Ginkgo biloba* leaf extract, the reduction of Ag^+^ ions to Ag^0^ was influenced by changing the silver nitrate concentration. A well-dispersed Ag-NP colloid was obtained at a lower silver nitrate concentration (0.02 M) of pH = 8. The average size of Ag-NPs measured using TEM is between 10 and 16 nm. The Ag-NPs prepared have strong antibacterial activity against both *Staphylococcus aureus* and *Escherichia coli* bacteria. The optimum conditions to prepare Ag-NPs using *Tinospora cordifolia* leaf extract studied by Selvam et al. (2017) occurred at 1.25 M of silver nitrate, 15 h of incubation time, 45°C of temperature and pH 4.5. The size of Ag-NPs determined using Scherrer's equation is 30 nm. The Ag-NPs are susceptible to *Klebsiella* and *Staphylococcus* bacteria species, showing a maximum zone of inhibition of 12.3 and 13.0 mm, respectively, at 10 mg/L of Ag-NPs.

In this study, a comparative study of the effect of physicochemical parameters to synthesis the Ag-NPs using aqueous extract stem extract of *E. spiralis* was performed, with the aim to find the superior parameter conditions for the biosynthesis of Ag-NPs with good properties of Ag-NPs. The effects of different physicochemical parameters studied include the initial concentration of silver nitrate, *E. spiralis* dosage, and stirring reaction time. The properties of Ag-NPs produced by aqueous extract stem extract of *E. spiralis* biosynthesis were characterized using UV-visible, XRD, FETEM, SEM, EDX, zeta potential, and FTIR techniques. The antibacterial activity of Ag-NPs performance was studied based on an antibacterial disk diffusion assay, in order to find the potential for both industrial and medical applications. Four bacteria from Gram-positive and Gram-negative bacteria including *Staphylococcus aureus* (ATCC 29523), *Enterococcus faecalis* (ATCC 29212), *Escherichia coli* (ATCC 25922), and *Proteus vulgaris* (ATCC 33420) were selected in this study.

## Materials and Methods

### Materials

The *E. spiralis* stem was collected from the forest in Tasik Chini, Pahang, Malaysia before being chopped and grounded to obtain *E. spiralis* stem powder. The silver nitrate was purchased from Acros organic, USA and was used without any purification. The deionized water from the ELGA Lab-Water/VWS (UK) purification system was used throughout the experiment. Four species of bacteria, including two Gram-positive species *Staphylococcus aureus* (*S. aureus*) (ATCC 25923) and *Enterococcus faecalis* (*E. faecalis*) (ATCC 29212) as well as two Gram-negative species *Escherichia coli* (*E. coli*) (ATCC 25922) and *Proteus vulgaris* (*P. vulgaris*) (ATCC 33420) were bought from Choice Care Sdn. Bhd, Kuala Lumpur, Malaysia. The stock culture was prepared in the Mueller Hinton broth (Difco, Malaysia) and incubated at 37°C overnight. For further usage, the stock culture was kept in the refrigerator at a temperature of 4–8°C.

### Preparation of *E. spiralis* Stem Aqueous Extraction

The aqueous extraction of the *E. spiralis* stem powder was done using the hot percolation method. A ratio of 1.5 g: 150 mL between *E. spiralis* dosage and deionized water was used. The mixture was stirred using a magnetic stirrer for 30 min. The temperature was then set at ~55°C for this extraction process. After stirring, the mixture was filtered and cooled before being used to synthesize Ag-NPs. The aqueous extract stem extract of *E. spiralis* is abbreviated as *E. spiralis* extract hereafter.

### Biosynthesis of *E. spiralis*/Ag-NPs

In general, 15 mL of silver nitrate (AgNO_3_) solution and 150 mL of *E. spiralis* extract was added in an Erlenmeyer flask. The mixture was stirred at 400 rpm using a magnetic stirrer at a temperature of ~52°C. The aluminum foil was used to cover the flask used during the Ag-NPs biosynthesis process because of the light-sensitivity of Ag-NPs. The reduction of Ag^+^ ions to Ag° (Ag-NPs) was observed preliminarily, based on its color when it changed to brown. The *E. spiralis*/Ag-NPs formation was then established using an UV-visible spectroscopy analysis. The effects of physicochemical parameters such as the initial concentration of AgNO_3_, *E. spiralis* dosage, and stirring reaction time, which have a significant impact on the shape, size, and distribution of *E. spiralis*/Ag-NPs, were studied. All the Ag-NPs biosynthesis experiments were conducted in batch mode and duplicated. In the end, the average results were reported as explained in the details methods below.

### Effect of Initial AgNO_3_ Concentrations

For the effect of initial concentrations of AgNO_3_, four different concentrations of AgNO_3_ were chosen (0.005, 0.050, 0.010, and 0.100 M). A mixture of 0.005 M AgNO_3_ (15 mL) and *E. spiralis* extract (1.5 g: 150 mL) was added in an Erlenmeyer flask and stirred at 400 rpm using a magnetic stirrer at ~52°C. During the Ag-NPs synthesis process, 10 mL of *E. spiralis*/Ag-NO_3_ solution was taken using a pipette after 15, 30, 60, 90, 120, 180, 240, 360, 480, and 600 min. All of the solutions were then put in a vial sample and kept at 4°C for further characterization studies. The aluminum foil was used to cover the flask used during the Ag-NPs biosynthesis process because of the light-sensitivity of Ag-NPs. The reduction of Ag^+^ ions to Ag^0^ was then analyzed using an UV-visible spectrophotometer (UV-vis 1800, Shimadzu). The color changes of the solution, after the formation of Ag-NPs, were also observed. The same procedure was then repeated for the initial AgNO_3_ concentration at 0.050, 0.010, and 0.100 M.

### Effect of *E. spiralis* Dosage

Regarding the effect of *E. spiralis* dosage, three different *E. spiralis* dosages were studied (0.5, 1.5, and 2.5 g). A volume of 15 mL AgNO_3_ (best concentration) and 150 mL of *E. spiralis* extract (0.5 g) was added in an Erlenmeyer flask. The mixture was stirred using a magnetic stirrer at 400 rpm and a temperature of ~52°C. During the Ag-NPs synthesis process, 10 mL of *E. spiralis*/Ag-NO_3_ solution was taken, using a pipette, after 15, 30, 60, 90, 120, 180, 240, 360, 480, and 600 min. All of the solutions were then put in a vial sample and kept at 4°C for further characterization studies. The aluminum foil was used to cover the flask used during the Ag-NPs biosynthesis process because of the light-sensitivity of Ag-NPs. The reduction of Ag^+^ ions to Ag^0^ was then analyzed using an UV-visible spectrophotometer. The color changes of the solution, after producing Ag-NPs, were also observed. The same procedure was then repeated to study the biosynthesis of Ag-NPs at an *E. spiralis* stem powder dosage of 1.5 and 2.5 g.

### Effect of Stirring Reaction Time

The study of the effect of reaction time after 600 min was extended to 720, 840, and 1,440 min. A volume of 15 mL AgNO_3_ (best concentration) and 150 mL of *E. spiralis* extract (best *E. spiralis* dosage) was added in an Erlenmeyer flask. The mixture was stirred using a magnetic stirrer at 400 rpm and a temperature of ~52°C. During the Ag-NPs synthesis process, 10 mL of *E. spiralis*/Ag-NO3 solution was taken, using a pipette, after 15, 30, 60, 90, 120, 180, 240, 360, 480, 600, 720, 840, and 1,440 min. All of the solutions were then put in a vial sample and kept at 4°C for further characterization studies. The aluminum foil was used to cover the flask used during the Ag-NPs biosynthesis process because of the light-sensitivity of Ag-NPs. The reduction of Ag^+^ ions to Ag^0^ was then analyzed using an UV-visible spectrophotometer. The color changes of the solution, after producing *E. spiralis*/Ag-NPs, were also observed.

### Characterization of *E. spiralis*/Ag-NPs

The synthesis of Ag-NPs was evaluated using an UV-vis spectroscopy analysis. This important characterization can produce crucial information on the shape, size, and distribution of Ag-NPs. The Ag-NPs solution was scanned from 300 to 700 nm with a UV-vis spectrophotometer at a medium rate. The crystalline structures of *E. spiralis*/Ag-NPs were determined using the X-ray diffractometer (XRD) (PANalytical X'pert PRO, Netherland) at 45 kV and a current of 30 mA with Cu-Kα radiation. The XRD pattern was initiated to scan from 10 to 90° at a 2θ angle. Only a selected sample was chosen for the XRD analysis of this study. The XRD sample of *E. spiralis*/Ag-NPs was ready with dried *E. spiralis*/Ag-NPs solution on the glass surface. The size and distribution of *E. spiralis*/Ag-NPs were investigated using the Field Emission Transmission Electron Microscope (FETEM) (JEOL, JEM-2100F, Japan). The stability of *E. spiralis/Ag-NPs* was determined using a zeta size analyzer (SZ-100, Horiba Scientific, Japan). The plausible mechanisms between Ag-NPs and the functional groups present in the *E. spiralis* extract, were predicted using the Fourier Transform Infrared (FTIR) spectrometer (Perkin Elmer, Frontier, USA) using the potassium bromide (KBr) pellet technique. The sample was scanned from the 4,000 to 400 cm^−1^ wavenumber.

### Antibacterial Disk Diffusion Assay

The antibacterial disk diffusion assay on antibacterial activity of *E. spiralis*/Ag-NPs was evaluated using the Kirby-Bauer technique (Bauer et al., 1966), which conformed to the recommended standards of the Clinical and Laboratory Standards Institute (CLSI). Two Gram-positive bacteria (*Staphylococcus aureus* ATCC 25922 and *Enterococcus faecalis* ATCC 29212) and two Gram-negative bacteria (*Escherichia coli* ATCC 25923 and *Proteus vulgaris* ATCC 33420) were used in this study. Gentamicin (10 μg/mL), plain disk and *E. spiralis* extract were used as positive and negative controls, respectively. The agar plate was prepared by pouring 20 mL of liquid Mueller Hinton agar (MHA) onto disposable sterilized petri dishes. The liquid MHA were allowed to solidify before being stored in the refrigerator at 4°C for further use. The bacteria suspension was prepared by subculturing 100 μL of stock culture bacteria into new Mueller Hinton broth (MHB). The bacteria suspension was incubated overnight at 37°C in the incubator. After incubation, the optical density OD_600_ of the bacterial suspension was adjusted to 0.1 absorbance, using UV spectrophotometer. This OD value corresponds to 1.5 × 10^6^ CFU/mL. The inoculum was then spread evenly over the MHA plate using a sterile cotton bud before applying the *E. spiralis*/Ag-NPs loaded disks. The experiment was carried out in triplicate and the diameter of the inhibition zone was measured after 24 h of incubation at 37°C. The results were recorded as the mean ± standard deviation of the triplicate experiment. All the data of antibacterial analysis were represented as the mean ± standard deviation of triplicate experiments. Sample *t*-test and one-way ANOVA was used to compare the statistical difference between two groups. The statistical data analysis was analyzed using SPSS version 22. A *p* < 0.05 was considered significant.

## Results

**Figure 1:**
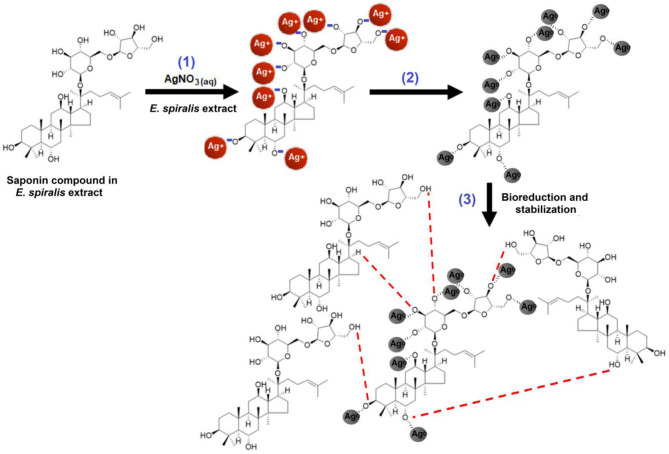
The possible mechanism between the reducing agents in the *E. spiralis* extract to reduce Ag^+^ ions to *E. spiralis*/Ag-NPs.

A preliminary tool was used to confirm the ability of *E. spiralis* extract to biosynthesize Ag-NPs, by observing the appearance of the signatory brown color of the solution. The reduction of Ag^+^ ions to Ag^0^ after being mixed with *E. spiralis* extract changed the color to dark brown. This color change confirmed the formation of Ag-NPs. In the *E. spiralis* extract, it contains the phytochemical compounds of terpenoid saponin and glycoside (Harun et al., 2015, 2016). Both phytochemical compounds are able to act as a reducing agent due to the presence of carboxyl and hydroxyl groups. According to Joy Prabu and Johnson (2015), these functional groups can reduce Ag^+^ ions to Ag^0^ by donating electron and hydrogen atoms that are then stabilized by the anionic functional groups in the *E. spiralis* extract. The schematic illustration of the suggested mechanisms of saponin compound presence in *E. spiralis* extract as a reducing and stabilizing agent to synthesize Ag-NPs is shown in Figure 1. In Figure 1, step (1) shows the hydroxyl groups (-OH) in the saponin compound were ionized to negatively charge –O^−^ groups in the aqueous medium, while AgNO_3_ was dissociated into Ag^+^ ions. The negatively charged O^−^ groups can attract electrostatically with the positively charged Ag^+^ ions. Step (2) shows that the reduction of Ag^+^ ions to Ag^0^ occurred by donating an electron and a hydrogen atom from the hydroxyl groups of the saponin compound which was then stabilized by the negatively charged functional groups in the saponin compound. Step (3) shows that the stabilization process then occurred due to the presence of van der Walls forces between the oxygen and negatively charged functional groups that surround the surface of Ag-NPs. This stabilization process controls the smaller size Ag-NPs in the synthesis process. This process is in agreement with Aramwit et al. (2014), who suggested that the hydroxyl groups are supposed to prevent their aggregation of Ag-NPs.

**Figure 2:**
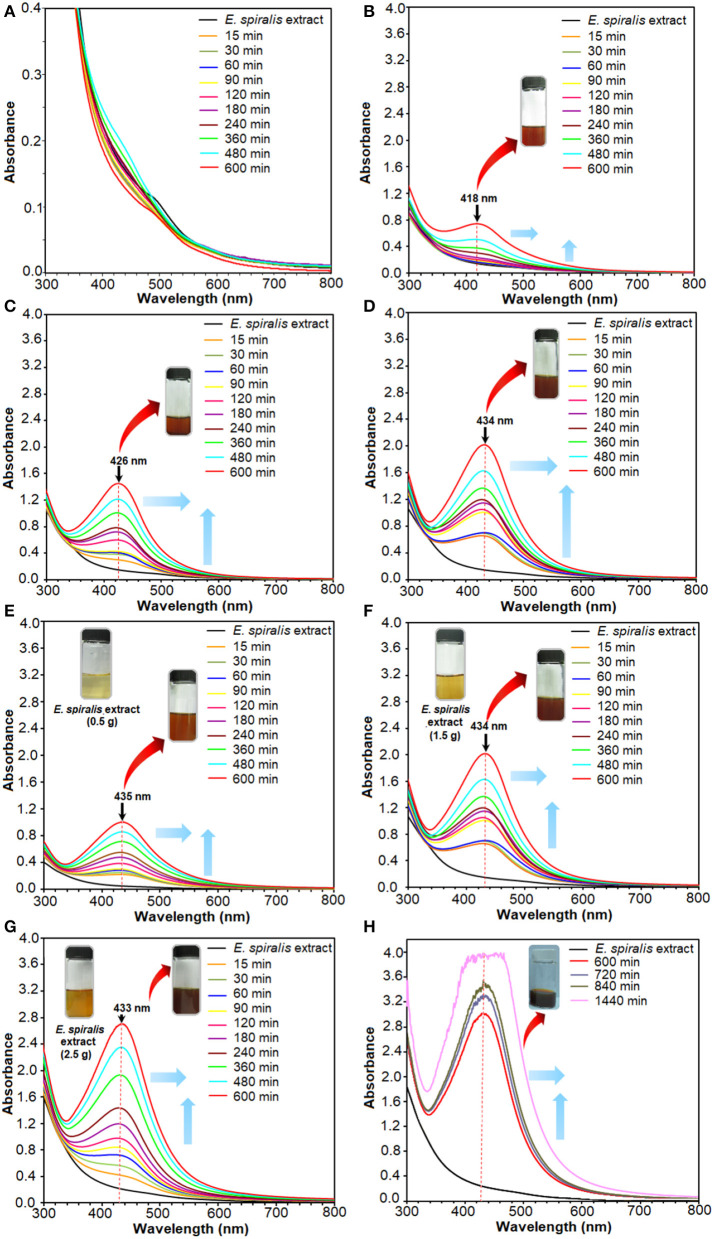
The UV-vis spectra of *E. spiralis*/Ag-NPs after the reaction at different initial concentrations of 0.005, 0.010, 0.050, and 0.100 M AgNO_3_ **(A–D)**, *E. spiralis* dosage of 0.5, 1.5, and 2.5 g **(E–G)**, and stirring reaction times **(H)**.

The solution also changed to a brown color after the reaction of Ag^+^ ions with *E. spiralis* extract at different initial concentrations of AgNO_3_ (0.005–0.100 M), *E. spiralis* dosage (0.5–2.5 g), and stirring reaction times (600–1,440 min) are shown in Figure 2. The figures show that the intensity of the brown color formation after the reaction with AgNO_3_ and *E. spiralis* extract was increased with the increase of the initial concentrations of AgNO_3_ from 0.005 to 0.100 M AgNO_3_ (Figures 2A–D), respectively. The same color changes observation was seen for the effect of *E. spiralis* stem powder dosage and stirring reaction times. It showed that the intensity of the brown color increased with the increase of the *E. spiralis* stem powder dosage from 0.5 to 2.5 g and the reaction times from 600 to 1,440 min as shown in Figures 2E–H, respectively. The different brown colors observed at all parameters is due to the excitation of the surface Plasmon resonance of Ag-NPs at different properties of Ag-NPs. The details of the studies will be reported in the UV-vis spectroscopy analysis. The same dark brown color changes, than the ability of Ag-NPs formation using *Cinnamomum tsoi* aqueous leaf extract, was reported by Maddinedi et al. (2017).

### UV-vis Spectroscopy Analysis

The UV-vis spectra can provide valuable information on the shape, size, and distribution of nanoparticles based on Surface Plasmon Resonance (SPR) bands. For instance, the appearance of the Ag peak at a shorter wavelength in the UV-vis spectra reveals the small size of Ag-NPs that were formed, while a longer wavelength indicates bigger Ag-NPs (Mashwani et al., 2016). These SPR bands arise from the interactions between the electron cloud on the Ag-NPs surface and the incoming electromagnetic radiation (Labulo et al., 2016). SPR bands centered between 420 and 430 nm of UV–vis absorption spectra correspond to the peak of Ag. The estimated size of Ag-NPs at these SPR bands range from 10 to 30 nm with a spherical shape (Sowmyya and Lakshmi, 2018).

The plant mediated synthesis of Ag-NPs using *E. spiralis* extract, as a reducing and stabilizing agent, was monitored by applying different initial concentrations of AgNO_3_, *E. spiralis* stem powder dosage, and stirring reaction times. From these results, the best parameter to synthesize Ag-NPs was chosen for further studies and characterization. The UV-vis spectra of *E. spiralis*/Ag-NPs at different initial concentrations of AgNO_3_ from 0.005 to 0.100 M and different stirring reaction times are shown in Figures 2A–D, respectively. From the results, it was found that the intensity of *E. spiralis*/Ag-NPs increases with increasing initial concentrations of Ag-NPs and reaction times. The initial concentration of AgNO_3_ should be more than 0.005 M to synthesize Ag-NPs using *E. spiralis* extract. A concentration of Ag^+^ ions below 0.005 M, will not be enough to reduce Ag° using *E. spiralis* extract as the reducing and stabilizing agent (Morales-Luckie et al., 2016). At 0.005 M AgNO_3_, no silver peaks appeared between 400 and 430 nm as shown in Figure 2A. The wavelength bands at 600 min of stirring reaction time were shifted to the larger wavelength (red shift) from 418 to 433 nm at 0.010 to 0.100 M AgNO_3_, respectively, as shown in Figures 2B–D. The intensity (absorbance) of *E. spiralis*/Ag-NPs increased from 0.745 to 2.020, respectively. According to Kumar et al. (2018), the larger wavelength of UV-vis indicates that a big sized nanoparticle was formed. The big size of the nanoparticles that was obtained when increasing the initial concentrations of AgNO_3_ might be due to the number of Ag-NPs being increased and which tend to aggregate each other. The increasing size of *E. spiralis*/Ag-NPs, based on the increase in the initial concentrations of AgNO_3_, was confirmed using the FETEM analysis. In this study, 0.100 M of AgNO_3_ at 600 min of reaction time was chosen as the best parameter for further study, considering the highest intensity and more sharpened peaks recorded at this concentration.

The results for the effect of *E. spiralis* dosage from 0.5 to 2.5 g at different stirring reaction times are shown in Figures 2E,F. In these figures, the wavelength bands at 600 min were shifted to a short wavelength (blue shift) when increasing the *E. spiralis* dosage from 435 to 433 nm at 0.5 to 2.5 g, respectively. This trend provides information on the smaller size of *E. spiralis*/Ag-NPs that have been formed. The decreased size of *E. spiralis*/Ag-NPs when increasing the *E. spiralis* dosage might be due to the increase of biomolecules (functional groups) that are present in the *E. spiralis* extract, which is able to stabilize the *E. spiralis*/Ag-NPs. The deceasing size of *E. spiralis*/Ag-NPs when increasing the *E. spiralis* dosage was supported by the FETEM analysis. The absorbance intensity of *E. spiralis*/Ag-NPs also increased from 1.005 to 2.703 when increasing the *E. spiralis* dosage. It has been predicted that the number of nanoparticles was increased (Labulo et al., 2016). Therefore, the best *E. spiralis* dosage of 2.5 g at 600 min of reaction time was chosen for further study and characterization.

The subsequent experiment was conducted using a constant value of 0.100 M AgNO_3_ and 2.5 g of *E. spiralis* stem powder dosage but the reaction times were increased at 720, 840, and 1,440 min. This experiment was conducted to ensure the best reaction time occurred at 600 min and the result is shown in Figure 2H. From the result, it shows that at 720 min, the UV- vis spectrum starts to become noise peaks, suggesting that the agglomeration of *E. spiralis*/Ag-NPs start to occur due to the big size of *E. spiralis*/Ag-NPs that has been formed. The wavelengths are moved to the red-shift (larger wavelength) indicating that the size of *E. spiralis*/Ag-NPs was increased when increasing the stirring reaction time. The peak intensity also increased with reaction time. These indicated an increase in the amount of Ag-NPs and a decrease in the number of Ag^+^ ions in the solution (Zhang et al., 2017). Therefore, the reaction time at 600 min was chosen as the reaction time to synthesize *E. spiralis*/Ag-NPs. The bigger size of Ag-NPs might reduce the performance of Ag-NPs for medical application.

### XRD Analysis

**Figure 3:**
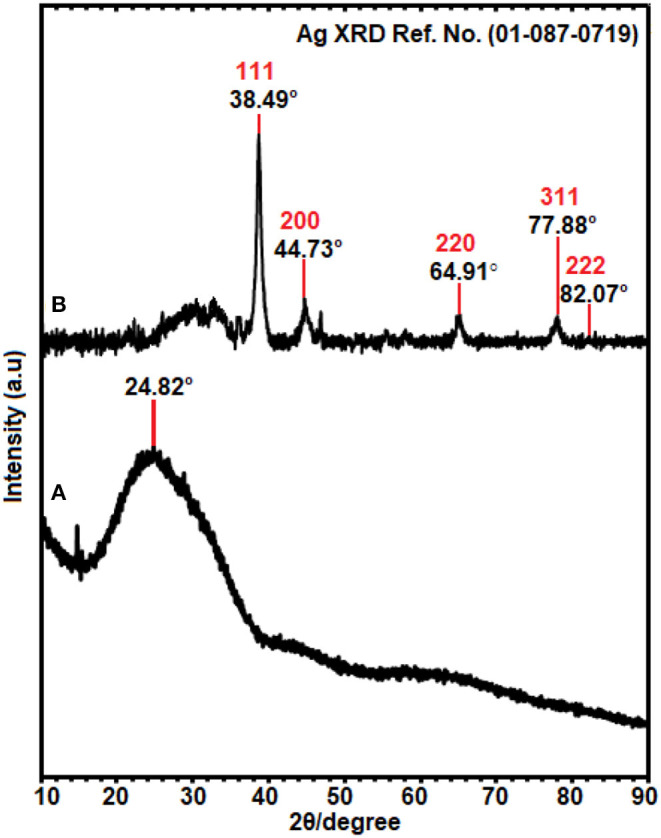
The XRD patterns of **(A)** *E. spiralis* extract (2.5 g) and **(B)** *E. spiralis*/Ag-NPs at the best parameters to synthesize Ag-NPs.

The crystallinity of Ag-NPs formed after synthesis, using *E. spiralis* extract, can be confirmed based on the XRD pattern. In this study, the sample of *E. spiralis*/Ag-NPs was determined only at the best physicochemical conditions (0.100 M AgNO_3_; 2.5 g *E. spiralis* stem powder dosage and 600 min of stirring reaction time). The XRD peaks of *E. spiralis* extract show only amorphous peaks at 24.82° with no silver peaks appearing, as shown in Figure 3A. In Figure 3B, the XRD pattern of *E. spiralis*/Ag-NPs shows the appearance of five diffractions peaks at 38.49°, 44.73°, 64.91°, 77.88°, and 82.07° at 2θ values. This XRD pattern was fitted with the indexed Face Center Cubic (FCC) at (111), (200), (220), and (311) of crystallographic planes of silver peaks (Ref. No. 01-087-0719), thereby confirming the crystalline properties of *E. spiralis*/Ag-NPs in nature. The crystallographic plane of silver data obtained was then further analyzed using SAED pattern and lattice spacing to further confirm the crystalline nature of *E. spiralis*/Ag-NPs.

### FETEM Analysis

**Figure 4:**
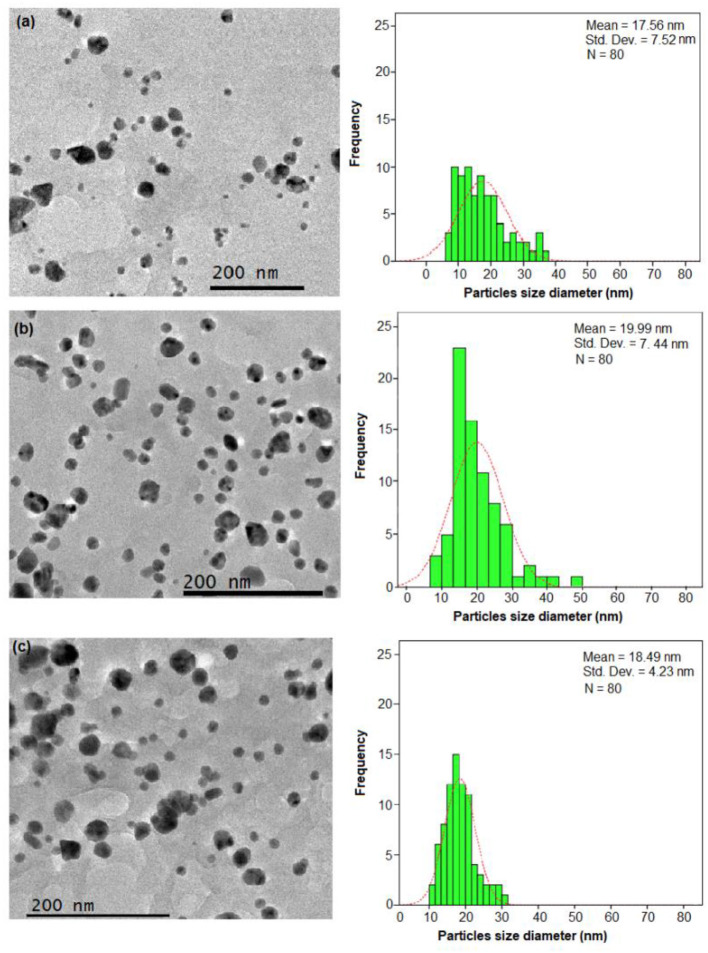
The FETEM images and histogram of the particle size distribution of *E. spiralis*/Ag-NPs at **(a)** 0.010 M of AgNO_3_ and **(b)** 0.100 M of AgNO_3_ and **(c)** at its best parameter condition, at 600 min of stirring reaction time.

The FETEM analysis is considered as the most important analysis in nanoparticle characterization studies. This analysis is useful to determine the shape, size, and morphology of Ag-NPs. In general, the *E. spiralis*/Ag-NPs showed mostly a spherical shape with the average particle size ranging from 18.15 ± 9.35 to 19.99 ± 7.44 nm, without significant agglomeration. The size, morphology, and distribution of *E. spiralis*/Ag-NPs at different initial concentrations of AgNO_3_ are shown in Figure 3. For the effect of initial concentrations of AgNO_3_, the average size of particles increased from 17.56 ± 7.52 to 19.99 ± 7.44 nm when increasing initial concentrations of AgNO_3_ from 0.010 to 0.100 M as shown in Figures 4a,b, respectively. This observation could be due to the increase in the amount of *E. spiralis*/Ag-NPs formed in the solution (Paosen et al., 2017). The increased number of nanoparticles present in the solution can cause agglomeration or cluster nanoparticles, thus increasing the size of nanoparticles. The results obtained are in line with the result of the UV-vis analysis which shows that the UV-vis spectra move to the red-shift, suggesting that the size of Ag-NPs increased when increasing initial concentrations of AgNO_3_.

For the effect of *E. spiralis* stem powder dosage, the average size of the nanoparticles decreased when increasing *E. spiralis* powder dosage from 1.5 to 2.5 g. The average size of *E spiralis*/Ag-NPs decreased from 19.99 ± 7.44 to 18.89 ± 4.75 nm (figures not shown). These findings proved the important role of *E. spiralis* extract to control the size of Ag-NPs by stabilizing it with the functional groups of *E. spiralis* extract. The increased amount of *E. spiralis* dosage also increases the available functional groups of *E. spiralis* extract and prevents further growth of *E. spiralis*/Ag-NPs. As such, the size of *E. spiralis/*Ag-NPs was decreased. This is because Ag-NPs are easy to agglomerate during the biosynthesis process due to the high tendency of silver nuclei to bond with Ag-NPs and the high surface area of the Ag-NPs (Polte, 2015). The decreasing number of *E. spiralis*/Ag-NPs at a 2.5 g dosage was chosen as the best parameter for further biomedical application, as shown in Figure 4c.

For the effect of stirring, reaction times also show that the size of *E. spiralis*/Ag-NPs slightly increased with the increase of reaction time at 60, 180, 360, and 600 min. The average size of *E. spiralis*/Ag-NPs increased slightly from 18.15 ± 9.35, 18.31 ± 6.83, 18.38 ± 5.15, and 18.89 ± 4.75 nm for 60, 180, 360, and 600 min, respectively. These results are in line with the UV-vis analysis, showing that wavelength moves to the red-shift, indicating the increased size of *E. spiralis*/Ag-NPs in the solution.

Overall, FETEM results showed that the average size of *E. spiralis*/Ag-NPs synthesized is <20 nm and is spherical in shape for all physicochemical parameters studied. This finding also supports the UV-vis results mentioned, which show that SPR bands centered between 420 and 430 nm estimated the spherical nanoparticles size range from 10 to 30 nm (Sowmyya and Lakshmi, 2018). According to Zhang et al. (2017), the diameters of the average particles from 5 to 50 nm have been shown to have strong antibacterial activity. The positive aspects of the antibacterial properties of *E. spiralis*/Ag-NPs was discussed in detail in the antibacterial activity section. From the FETEM results, it shows that Ag-NPs were successfully synthesized using *E. spiralis* extract.

**Figure 5:**
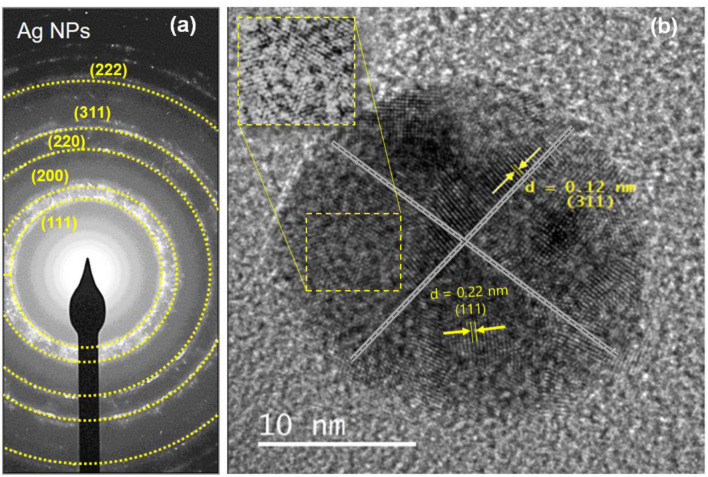
The SAED pattern of **(a)** *E. spiralis*/Ag-NPs and **(b)** the lattice spacing of *E. spiralis*/Ag-NPs.

Figure 5 shows the results of the selective area electron diffraction (SAED) pattern at the best parameters. The rings around the SAED pattern are in line with the XRD pattern as shown in Figure 5a. The pattern corresponding to the cubic Ag-NPs planes of (111), (200), (220), (311), and (222), qualified the polycrystalline nature of *E. spiralis*/Ag-NPs. In Figure 5b, the FETEM image of an individual Ag-NPs with a lattice spacing of ~0.22 and 0.12 nm correspond to the d spacing of the (111) and (311) cubic plains of Ag-NPs at 38.49° and 77.88° angles, which are consistent with the XRD peak as shown in Figure 3B.

**Figure 6:**
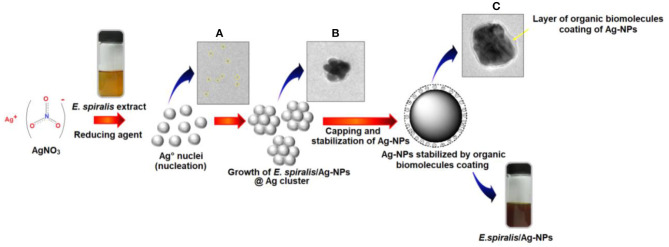
Bottom to top approaches of *E. spiralis*/Ag-NPs formation. **(A)** Formation of Ago nuclei. **(B)** Growth of *E. spiralis*/Ag-NPs. **(C)** Stabilization of Ag-NPs by organic biomolecules of *E. spiralis* extract.

The FETEM analysis is also useful to predict the possible mechanism of *E. spiralis*/Ag-NPs mediated by *E. spiralis* extract, as illustrated in Figure 6. The mechanisms are related to bottom-up approaches starting with a chemical reaction by the self-assembly of atoms to new nuclei which grow into nanoparticles (Polte, 2015; Ahmed et al., 2016). The *E. spiralis*/Ag-NPs are prepared in aqueous solution by the reduction of a dissolved metal precursor of AgNO_3_, using the reducing agent in the *E. spiralis* extract. The *E. spiralis* extract also acts as a stabilizing agent that controls the Ag-NPs from aggregation or clustering. The stabilizing agent is negatively surface charged and is able to be attached on the positively surface charged Ag-NPs, thus providing the repulsive forces of electrostatic stabilization that can suppress Ag-NPs from aggregation (Banach and Pulit-Prociak, 2017). The negative surface charges of *E. spiralis*/Ag-NPs are confirmed by the zeta potential analysis section. The micrograph image of Ag° nuclei, growth *E. spiralis*/Ag-NPs, and *E. spiralis*/Ag-NPs, stabilized by biomolecules in the *E. spiralis* extract, are shown in Figures 6A–C, respectively.

### Zeta Potential Analysis

**Figure 7:**
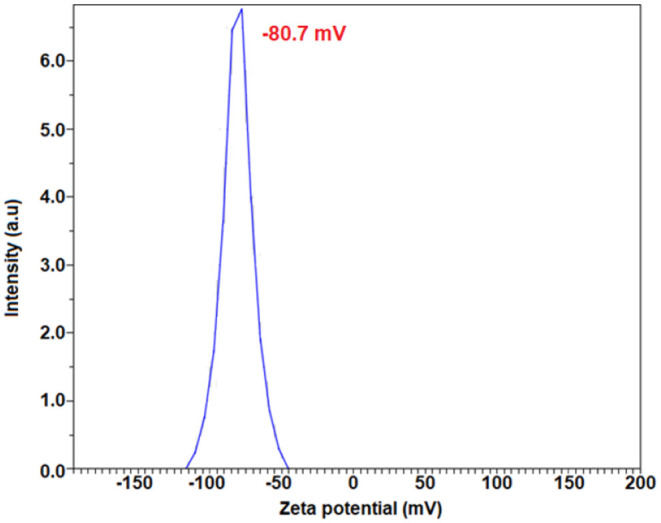
The zeta potential result of *E. spiralis* extract at 2.5 g *E. spiralis* stem powder dosage.

The surface properties and stability of the *E. spiralis* extract and *E. spiralis*/Ag-NPs can be determined using zeta potential analysis. In general, all the samples have shown the negative value of zeta potential. According to Faried et al. (2016), the negative surface value (more than 30 mV) shows the stability of the *E. spiralis*/Ag-NPs colloid. The previous study, reporting on biosynthesized silver nanoparticles using other plant extracts, is summarized in Table 1. It was found that the big size and low stability of Ag-NPs have been synthesized. In this study, the value of zeta potential for *E. spiralis* extract at 2.5 g of *E. spiralis* dosage is −80.7 mV, as shown in Figure 7. This value shows that the high stability of *E. spiralis* extracts to synthesize Ag-NPs. The negative value of the zeta potential record suggests that the negative surface charge of *E. spiralis* extracts might come from the OH^−^, COO^−^, CO^−^ functional groups. The detailed discussion of these functional groups and its possible mechanisms were discussed in detail in the FTIR analysis. The stability of Ag-NPs causes difficulties to agglomerate and increases the performance of Ag-NPs. The zeta potential value of *E. spiralis*/Ag-NPs at different reaction times is decreased with increased reaction time. The zeta potential value at 60, 180, 360, and 600 min, using 2.5 g *E. spiralis* dosage, are; 72.8, −74.4, −78.0, and −83.4 mV, respectively. The high negative zeta potential value might be due to the coordination of anionic stabilizing agents in *E. spiralis* extract with the Ag-NPs. A negatively charged Ag-NPs surface prevented the nanoparticles from aggregation and stabilized Ag-NPs by the electrostatic repulsions among the negative charges (Paosen et al., 2017).

**Table 1:** Summary of the synthesis of Ag-NPs using other plant extracts.

**Plant**	**Size and shape of Ag-NPs**	**Zeta potential value (mV)**	**References**
*Soymida febrifuga* (stem bark)	10–30 nm; spherical	−34.7	Sowmyya and Lakshmi, 2018
*Sapindus mukorossi* and *Acacia concinna* (leaves)	30 nm; spherical	−50 to 55	Sur et al., 2018
*Enicostemma axillare* (leaves)	18 nm; spherical	−24.6	Raj et al., 2018
*Allium ampeloprasum* (leaves)	2–43 nm; quasi spherical, spherical, ellipsoidal, hexagonal and irregular	−15.1	Khoshnamvand et al., 2019
*Calliandra haematocephala* (leaves)	70 nm; spherical	−17.2	Raja et al., 2017

### FTIR Spectroscopy Analysis

**Figure 8:**
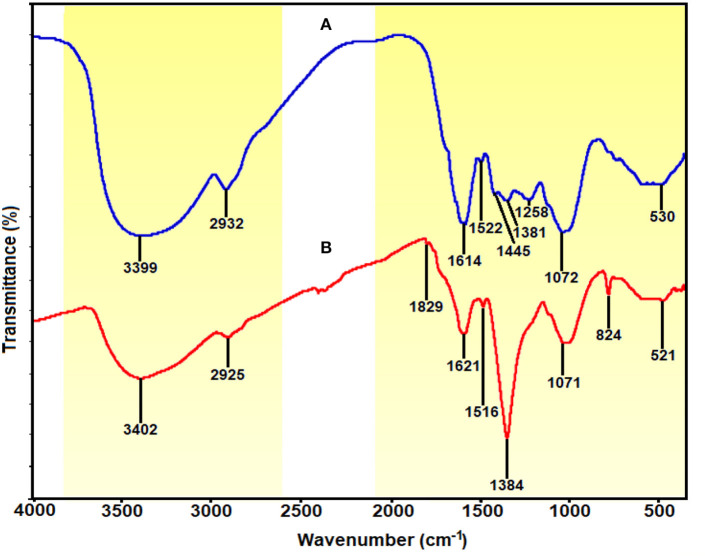
FTIR spectra of **(A)** *E. spiralis* extract at 2.5 g of *E. spiralis* dosage and **(B)** *E. spiralis*/Ag-NPs at best parameters of the biosynthesis process.

The shifting in wavenumber or changes in peak intensity explains the types of functional groups involved in the binding mechanisms. The FTIR spectrum for *E. spiralis* extract at 2.5 g of *E. spiralis* dosage showed the absorption bands at 3,399, 2,932, 1,614, 1,522, 1,445, 1,381, 1,258, 1,072, and 530 cm^−1^, as shown in Figure 8A. According to Pavia et al. (2009), the peaks ranging from 3,200 to 3,600 cm^−1^ are related to the O-H (hydroxyl) and -NH_2_ (amine) stretching vibrations in the *E. spiralis* extract. The peak at 2,932 cm^−1^ can be assigned to C-H stretching. The peak at 1,614 cm^−1^ represents N-H bending from the glycoside compound in *E. spiralis* extract. The peak at 1,522 cm^−1^ corresponds to the aromatic ring of the terpenoid saponin structure. The carboxylate group can be confirmed by the 1,445 cm^−1^. The peak at 1,381 cm^−1^ corresponds to the C-H bending of aldehyde groups from the glucose structure in *E. spiralis* extract. The peak at 1,258 cm^−1^ corresponds to the C-C(=O)-O stretching of ester (Silverstein et al., 1991). The peak at 1,072 cm^−1^ corresponds to C-O stretching. The peak at 530 cm^−1^ is related to the bonding of oxygen from the hydroxyl groups.

However, for the *E. spiralis*/Ag-NPs synthesized at best parameters, the FTIR spectrum showed some shifting of the peaks; peak intensity decreased/increased, and disappeared, as observed in Figure 8B. The peak shifted, suggesting that the responsible functional groups were involved in the binding mechanism on the Ag-NPs. After the synthesis process, the peaks at 3,399, 2,932, 1,522, and 530 cm^−1^ shifted to 3,402, 2,925, 1,516, and 521 cm^−1^ corresponding to O-H and –NH_2_ stretching, C=O, C-H stretching, the aromatic ring of the terpenoid structure, and Ag-O, respectively (Shameli et al., 2012; Banach and Pulit-Prociak, 2017). The appearance of a new peak at 1,829 cm^−1^ represented carboxylate and C=C in the aromatic groups from the terpenoid saponin structure. This peak confirmed that the glucose structure attached at terpenoid saponin as an aldehyde oxidize to gluconic acid. The new peak at 823 cm^−1^ also increased, suggesting that C-H groups might also be bonded with the Ag-NPs. The disappearance of some peaks at 1,445 and 1,258 cm^−1^ has been observed in the *E. spiralis*/Ag-NPs FTIR spectrum. These peaks suggested that the binding mechanisms of Ag-NPs with *E. spiralis* extract occurred at carboxylate groups and C-C(=O)-O stretching of the ester. The peak intensity at 1,621 cm^−1^ decreased, suggesting the involvement of N-H bending from the glycoside. However, the peak intensity at 1,384 cm^−1^ increased, proposing the C-H bending of aldehyde groups from the glucose structure in *E. spiralis* extract. All the peak changes support the impact of functional groups of *E. spiralis* extract as reducing and stabilizing agents to synthesize Ag-NPs.

### Antibacterial Activity of *E. spiralis*/Ag-NPs

**Figure 9:**
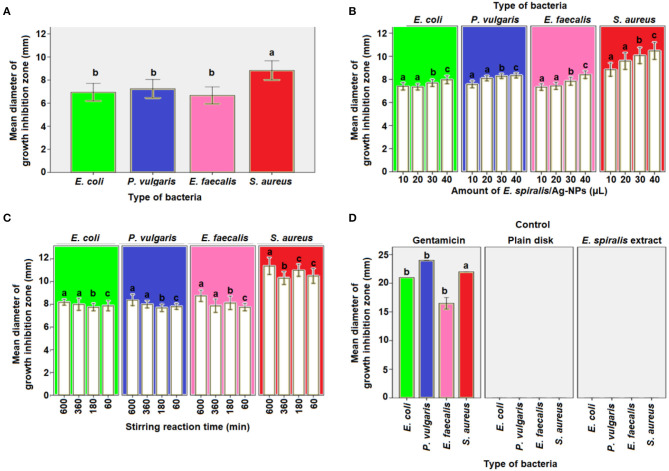
The mean diameter of inhibition zones by *E. spiralis*/Ag-NPs at **(A)** different type of bacteria, **(B)** different amount of *E. spiralis*/Ag-NPs against different type of bacteria, **(C)** different stirring reaction time against different type of bacteria, **(D)** positive and negative control of antibacterial agent. Data with the same letter are not significantly different (*P* > 0.05).

The antibacterial activity of *E. spiralis/Ag-NPs* was evaluated based on the diameter of the growth inhibition zone at different parameters and bacteria species, and the results are shown in Table 2. The order of the highest antibacterial activity is *S. aureus* > *P. vulgaris* > *E. coli* > *E. faecalis* species, respectively as observed in Figure 9A. There are significant differences in the diameter of the growth inhibition zone between the bacteria species based on the *post-hoc* multiple comparisons Tukey HSD test (*p* < 0.05). The significant difference in the diameter of the growth inhibition zone was observed between *S. aureus* and *E. faecalis, E. coli*, and *P. vulgaris*. However, the bacteria of *E. faecalis, E. coli*, and *P. vulgaris* did not significantly increase the diameter of the growth inhibition zone (*p* > 0.05). The weak antibacterial activity of *E. spiralis*/Ag-NPs, at best parameters, toward Gram-negative bacteria might be due to the material like capsule that has been covered on the bacteria cell wall and also the negatively charged outer lipid membrane (lipopolysaccharide) cover (Patil et al., 2018). The negative charge of both Gram-negative bacteria and *E. spiralis*/Ag-NPs caused the electrostatic repulsion between them, and as such hinders the attachment and penetration into the cells (Ahmad et al., 2017). The negative charge of *E. spiralis*/Ag-NPs is evident from the zeta potential value. Further analysis was conducted on the significant differences in antibacterial activity between Gram-positive and Gram-negative bacteria species. Surprisingly, the differences between these bacteria are not significant (*p* > 0.05). This result proved that the *E. spiralis*/Ag-NPs has the antibacterial activity for both types of Gram-positive and Gram-negative bacteria.

**Table 2:** The diameter of the growth inhibition zone at different parameters against different types of bacteria species.

**Bacteria species**	**Mean diameter of growth inhibition zone (mm)**			
	***E. coli***	***P. vulgaris***	***E. faecalis***	***S. aureus***
**Amount of** ***E. spiralis*****/Ag-NPs (μL)**				
10	7.40 ± 0.16	7.57 ± 0.16	7.33 ± 0.14	8.84 ± 0.29
20	7.34 ± 0.13	8.09 ± 0.11	7.94 ± 0.16	9.60, 0.36
30	7.69 ± 0.15	8.28 ± 0.10	7.84 ± 0.18	10.06, 0.35
40	7.97 ± 0.17	8.34 ± 0.11	8.41 ± 0.17	10.47, 0.37
**Stirring reaction time (min)**				
600	8.19 ± 0.13	8.38 ± 0.25	8.75 ± 0.25	11.38, 0.38
360	8.00 ± 0.27	8.00 ± 0.16	7.88 ± 0.30	10.31, 0.28
180	7.75 ± 0.16	7.78 ± 0.16	8.13 ± 0.30	11.00, 0.27
60	7.88 ± 0.23	7.81 ± 0.13	7.75 ± 0.16	10.50, 0.33
**Control**				
Gentamicin (+ve)	21.00 ± 0.00	24.00 ± 0.00	16.50 ± 0.50	22.00, 0.00
*E. spiralis* extract (–ve)	^*^NA	^*^NA	^*^NA	^*^NA
Plain disk (–ve)	^*^NA	^*^NA	^*^NA	^*^NA

* *NA means not available*;

*is mean ± SE of three experiment.

To investigate if the amount of *E. spiralis*/Ag-NPs has any effects on the antibacterial activity, different amounts of *E. spiralis*/Ag-NPs (μL), ranging from 10 to 40 μL, were used. The diameter of the growth inhibition zone was slightly increased when increasing the amount of *E. spiralis*/Ag-NPs from 10 to 40 μL, as shown in Table 2. This phenomenon can be explained because more Ag-NPs accumulated on the bacteria surface, entering the cell from inside, damaging the nuclei, and causing bacterial death (Deshmukh et al., 2019). There is a significant difference in the diameter of the growth inhibition zone between the amount of *E. spiralis*/Ag-NPs used (*p* < 0.05). The multiple comparison *post-hoc* test value shows no significant difference in the diameter of the growth inhibition zone between 10 and 20 μL (*p* > 0.05), however, there was a significant difference in the diameter of the growth inhibition zone between 30 and 40 μL (*p* < 0.05), as shown in Figure 9B. This explained dose-dependent antibacterial activity by *E. spiralis*/Ag-NPs.

The diameter of the growth inhibition zone at synthesized *E. spiralis*/Ag-NPs at different stirring reaction times, ranging from 60 to 600 min, is shown in Table 2. From the table, the diameter of the growth inhibition zone slightly increases when increasing the stirring reaction time. Most of the literature mentions that the antimicrobial activities are better in smaller nanoparticles (Alsammarraie et al., 2018), however, the average size of *E. spiralis*/Ag-NPs was slightly increased when increasing the stirring reaction time, as reported in the FETEM analysis. This phenomenon can be explained by the smaller-sized distribution of *E. spiralis*/Ag-NPs, with an increase in stirring reaction time, as observed in the FETEM analysis (Usman et al., 2013). Therefore, besides smaller sized nanoparticles, good distribution of nanoparticles should also be considered as another factor of positive antibacterial activities. However, there is no significant difference, when increasing the stirring reaction time, on the diameter of the growth inhibition zone (*p* > 0.05), as shown in Figure 9C. The positive control of the gentamicin antibiotic standard shows the significant differences (*p* < 0.05) between the negative control of *E. spiralis* extract and the plain disk (Figure 9D). The negative control of *E. spiralis* extract demonstrates that the antibacterial activity is due to Ag-NPs. According to Pollini et al. (2009), with reference to standard antibacterial test “SNV 195920-1992,” a the microbial zone of inhibition of more than 1.0 mm in diameter, can be considered to have potential with good antimicrobial activity. Thus, the *E. spiralis*/Ag-NPs can be considered as a good antibacterial agent for the application in biomedical and wastewater treatment.

**Figure 10:**
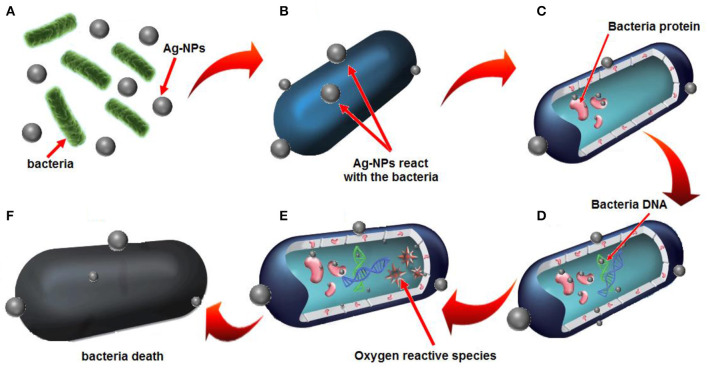
Plausible mechanisms of antibacterial activity by *E. spiralis*/Ag-NPs is based on **(A)** electrostatic attraction of bacteria with *E. spiralis*/Ag-NPs, **(B)** the release of Ag^+^ ions, **(C)** electrostatic attraction between Ag^+^ ions and bacteria protein, **(D)** the interference with bacterial DNA replication, **(E)** resulting in oxygen reactive species, **(F)** bacteria death.

The plausible mechanism of antibacterial activity of *E. spiralis*/Ag-NPs can be explained based on the electrostatic attraction between the negatively charged microorganism cell membrane and the Ag^+^ ion. Therefore, the Ag^+^ ion can interact with thiol groups of enzymes, destroys the DNA replication ability, followed by bacterial cell death (Li et al., 2016). The plausible mechanism of antibacterial activity by *E. spiralis*/Ag-NPs is depicted in Figure 10. According to Sabry et al. (2018), the possible mechanisms could be replaced by the contact action of Ag-NPs with the bacterial surface which will combine with the bacterial protein in the cell wall (Figures 10A–C). This can interfere with the DNA replication and also promote the generation of reactive oxygen species which will then cause bacterial cell death, as shown in Figure 10D.

## Conclusion

The developed method of the biosynthesis of Ag-NPs mediated by *E. spiralis* extract using an eco-friendly method, good distribution, and high percentage yield, was in fact successful. The effect of physicochemical parameters such as initial concentrations of AgNO_3_, *E. spiralis* dosage, and stirring reaction time has a significant effect on the properties of *E. spiralis*/Ag-NPs and the antimicrobial activity. The smaller size of *E. spiralis*/Ag-NPs was obtained at highest *E. spiralis* dosage, but the larger size of *E. spiralis*/Ag-NPs was obtained at the highest initial concentration of AgNO_3_. The formation of *E. spiralis*/Ag-NPs was confirmed by UV-vis spectra in the SPR bands ranging from 418 to 434 nm. The XRD analysis showed the crystalline structure of *E. spiralis*/Ag-NPs silver of FCC at (111), (200), (220), and (311). The FETEM analysis showed the spherical shape of *E. spiralis*/Ag-NPs with a good distribution of nanoparticles and the average size ranged from 18.15 ± 9.35 to 19.99 ± 7.44 nm. The SAED pattern was confirmed, with the XRD results showing that the lattice spacing of ~0.12 nm corresponds to the d spacing of the (311) cubic plain of Ag-NPs at an angle of 77.88°. The reducing and stabilizing agents might come from O-H and –NH_2_ stretching, C=O, C-H stretching, the aromatic ring of the terpenoid structure and Ag-O, hydroxyl groups (oxygen), carboxylate groups, and C-C(=O)-O stretching of the ester groups of *E. spiralis* extract, as revealed by FTIR analysis. The prepared Ag-NPs mediated by *E. spiralis* extract also exhibited excellent antimicrobial activity toward pathogenic bacteria namely *S. aureus, E. faecalis, E. coli*, and *P. vulgaris*. Thus, *E. spiralis*/Ag-NPs have potential as a promising nanomaterial for biomedical applications such as in wound healing and the coating of biomaterials.

## Data Availability Statement

All datasets generated for this study are included in the article/supplementary material.

## Author Contributions

WW: synthesis, experimental works, and writing original draft. KS: supervision, review, and editing. NC: dye catalytic analysis or interpretation of data, review, and editing. NO: sample characterization. NH: antibacterial analysis or interpretation of data, review, and editing. All authors contributed to the article and approved the submitted version.

## Conflict of Interest

The authors declare that the research was conducted in the absence of any commercial or financial relationships that could be construed as a potential conflict of interest.
